# Is Selenium a Potential Treatment for Cancer Metastasis?

**DOI:** 10.3390/nu5041149

**Published:** 2013-04-08

**Authors:** Yu-Chi Chen, K. Sandeep Prabhu, Andrea M. Mastro

**Affiliations:** 1 Department of Biochemistry and Molecular Cell Biology, The Pennsylvania State University, University Park, PA 16802, USA; E-Mail: yzc116@psu.edu; 2 Department of Veterinary and Biomedical Sciences, The Pennsylvania State University, University Park, PA 16802, USA; E-Mail: Ksp4@psu.edu; 3 Center for Molecular Toxicology and Carcinogenesis, The Pennsylvania State University, University Park, PA 16802, USA; 4 Center for Molecular Immunology and Infectious Disease, The Pennsylvania State University, University Park, PA 16802, USA

**Keywords:** selenium, selenoproteins, metastasis, migration, invasion, angiogenesis

## Abstract

Selenium (Se) is an essential micronutrient that functions as a redox gatekeeper through its incorporation into proteins to alleviate oxidative stress in cells. Although the epidemiological data are somewhat controversial, the results of many studies suggest that inorganic and organic forms of Se negatively affect cancer progression, and that several selenoproteins, such as GPXs, also play important roles in tumor development. Recently, a few scientists have examined the relationship between Se and metastasis, a late event in cancer progression, and have evaluated the potential of Se as an anti-angiogenesis or anti-metastasis agent. In this review, we present the current knowledge about Se compounds and selenoproteins, and their effects on the development of metastasis, with an emphasis on cell migration, invasion, and angiogenesis. In the cancers of breast, prostate, colorectal, fibrosarcoma, melanoma, liver, lung, oral squamous cell carcinoma, and brain glioma, there is either clinical evidence linking selenoproteins, such as thioredoxin reductase-1 to lymph node metastasis; *in vitro* studies indicating that Se compounds and selenoproteins inhibited cell motility, migration, and invasion, and reduced angiogenic factors in some of these cancer cells; or animal studies showing that Se supplementation resulted in reduced microvessel density and metastasis. Together, these data support the notion that Se may be an anti-metastastatic element in addition to being a cancer preventative agent.

## Abbreviations

Se: selenium
Sec: selenocysteine
SeMet: selenomethionine
MSC: Se-methyl-selenocysteine
SECIS: selenocysteine-insertion sequence
MeCN: methylselenocyanate
SELECT: the Selenium and Vitamin E Cancer Prevention Trial
GPX: glutathione peroxidase
TXR: thioredoxin reductase
SBP: selenium-binding protein
MSA: methylseleninic acid
VEGF: vascular endothelial growth factor
HGF: hepatocyte growth factor
IL-1: interlukin-1
IL-8: interlukin-8
SDF-1: stromal cell-derived factor 1
GRO-α: growth-regulated peptide-alpha/growth-regulated oncogene-1
OPN: osteopontin
FGF: fibroblast growth factors
MMP: matrix metalloproteinase
HUVEC: human umbilical vein endothelial cells
HIF-1α: hypoxia-induced factor 1alpha
SCID mice: severe combined immunodeficient mice
Sep15: the 15-KDa selenoprotein
uPA: urokinase-type plasminogen activator
ECM: extracellular matrix
TIMP1: tissue inhibitor of metalloproteinase 1
TIMP2: tissue inhibitor of metalloproteinase 2
PMA: 12-o-tetradecanoylphorbol-13-acetate
MT1-MMP: membrane-type 1 matrix metalloproteinase
IL-18: interlukin-18
PAI-1: plasminogen activator inhibitor-1
TNFα: tumor necrosis factor alpha
IGF II: insulin-like growth factor II
COX-2: cyclooxygenase-2
iNOS: inducible nitric oxide synthase

## 1. Introduction

Unlike other trace elements that interact with proteins non-covalently, selenium (Se) is an essential and unique micronutrient in that it is co-translationally incorporated into polypeptides in the form of the 21st amino acid, selenocysteine (Sec). Generally, selenoproteins can be classified into three categories. First, there are selenoproteins that have incorporated Sec under a precise process requiring the UGA codon, a specified tRNA (Sec tRNA ^[Ser]Sec^), some regulatory proteins, and the Sec-insertion sequence (SECIS) element [1]. The Sec residues in these selenoproteins are often located in the active site and are critical for their function. There are twenty five selenoproteins identified in the human genome so far [2,3]. The biological functions of some selenoproteins, including glutathione peroxidases (GPXs), deiodinases, and thioredoxin reductases (TXRs), have been studied extensively; while the functions of other selenoproteins, such as selenoprotein K, remain largely unknown. Second, there are proteins that contain selenomethionine (SeMet), in addition to Sec, as a result of their random substitution for cysteine and methionine due to the structural similarity between cysteine and Sec and between methionine and SeMet. Finally, the third class consists of selenium-binding proteins (SBP), which bind Se by some unknown mechanisms [4]. 

**Figure 1:**
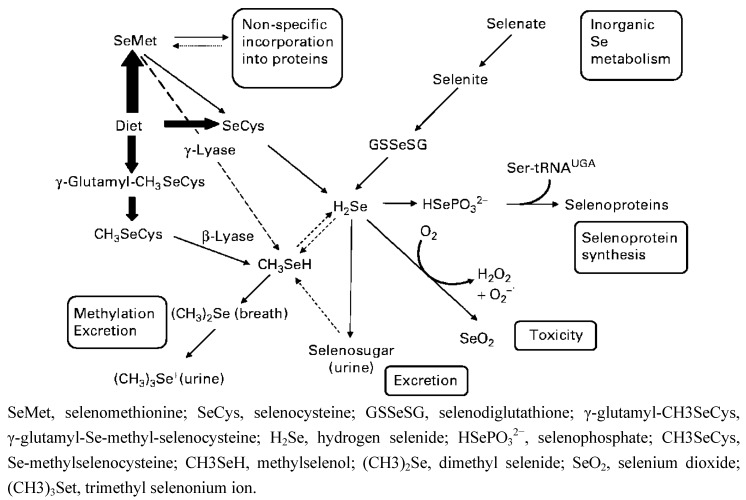
Metabolic pathway of dietary selenium (Se) in humans. Reproduced with permission from [5].

To synthesize Sec, cells have to process various Se compounds obtained from food in order to generate selenophosphate. Selenophosphate interacts with tRNA-bound seryl residues and forms a specific tRNA (Sec tRNA ^[Ser]Sec^). Several Se compounds abundant in plants and animals include SeMet, Sec, Se-methyl-selenocysteine (MSC), γ-glutamyl-Se-methyl-selenocysteine, selenate, and selenite. SeMet, Sec, selenite, and selenite can be converted to the common metabolite, hydrogen selenide (H_2_Se) that serves as the precursor of selenophosphate (Figure 1). In addition to SeMet, MSC and γ-glutamyl-Se-methyl-Sec also generate methylselenol (CH_3_SeH). The equilibrium between methylselenol and selenide ensures the use of methylselenol as a Se source when needed [5]. Other than the natural forms of Se, laboratory-synthesized Se compounds, such as methylseleninic acid (MSA), a precursor of methyselenol, have also been extensively studied for their possible therapeutic applications [6]. It is known that when cells generate too much selenide, it reacts with oxygen to produce superoxide, which is toxic to cells [7]. On the other hand, the anti-cancer effects of Se have been shown to involve methylselenol [7,8]. Taken together, various Se compounds may enter the metabolic pathway at different points catalyzed by different enzymes (Figure 1). More importantly, results of several studies show that some effects and/or mechanisms of Se are specific to certain forms of Se [9,10,11]. For instance, in LNCaP human prostate cancer cells, selenite was more effective in inducing apoptosis than MSA, while DU145 human prostate cancer cells were more sensitive to MSA-induced apoptosis [12]. In addition, due to the easy conversion to selenide, 5 ppm selenite is considered toxic [13]. On the other hand, it has been reported that humans can tolerate much higher SeMet supplementation (7200 μg twice daily for seven days and then a single dose daily for a few weeks) without side effects [13]. The high tolerance and low toxicity profile of organic Se allow the plasma Se to exceed 15 μM, the concentration required to enhance the efficacy and reduce toxicity when Se was used in combination with chemotherapeutic drugs in animal models [13]. Therefore, organic Se may have more potential as an anti-cancer therapeutic than inorganic Se. 

For decades, the benefit of Se supplementation on human health has been studied extensively. Primarily, Se supplementation has been considered to be an anti-oxidant endowed with anti-inflammatory and anti-viral activities [1]. In 1985, Clark *et al.* reported an inverse correlation between the Se content in forage crops in the United States and overall cancer mortality [14]. Supported by these findings, the Nutritional Prevention of Cancer Trial (NPC) was designed to evaluate the advantages of Se supplementation (as Se-enriched yeast) on the recurrence of non-melanoma skin cancer [15]. This trial is now best valued for its secondary findings, which indicated that dietary Se significantly reduced overall cancer mortality and the incidence of prostate, colorectal, lung, and total cancers in male participants during 1983–1993. However, only the reduction in the incidence of total and prostate cancers remained significant in a later analysis in 1996 [16]. It is also noted that Se supplementation had the greatest impact in men with the lowest Se baseline (<106.4 ng/mL in plasma) [15]. The positive results of the NPC trial and several other studies led to a large phase III chemoprevention trial, the Selenium and Vitamin E Cancer Prevention Trial (SELECT) with the expectation that Se and/or Vitamin E supplementation would effectively prevent prostate cancer [17]. However, the SELECT trial was terminated prematurely in 2008 due to the lack of a preventive effect. A recent follow-up study did not show any long term preventive potential either [18]. The criticisms, post-SELECT, did not discourage the studies and applications of Se to cancer research. Rather, it served as a “check-point” for researchers to review and modify strategies. For instance, the importance of baseline Se levels in participants has been evaluated [3]. Other than the studies of the effects on prostate cancer, results from other studies (performed on a smaller scale) suggested that Se supplementation exhibited beneficial effects on lung, bladder, colorectal, oesophageal, gastric cardia, and thyroid cancers [19,20,21,22,23]. Taken together, there is credible evidence of beneficial effects of Se supplementation in reducing cancer risks at least in certain subpopulations that have low levels of Se. 

Despite mixed and hard-to-interpret clinical data, the results of many *in vitro* and pre-clinical studies have demonstrated the anti-cancer and chemopreventive actions of Se [24,25,26]. In this review, we chose to emphasize the impact of Se on metastasis since the literature is replete with reviews that discuss the potential mechanisms through which Se can suppress tumor initiation and primary tumor growth [27,28]. Therefore, we will mainly focus on the role of Se in metastasis. Briefly, Se can affect cancer by regulating the expression of redox-active proteins, modulating the redox status of several proteins, balancing intracellular redox status, regulating inflammatory and immune responses, maintaining DNA stability, inducing cell cycle arrest and apoptosis, inhibiting local invasion and migration, blocking angiogenesis, activating or inactivating crucial regulatory proteins of cell proliferation, and enhancing phase II-carcinogen-detoxifying enzymes [26]. There are many reports in the literature presenting examples of the involvement of Se in the inhibition of carcinogenesis and in the treatment of localized tumors. However, much less research is focused on the later events of cancer progression and the development of metastasis. Given that metastasis is often the cause of death among cancer patients, it is surprising how little information is available on the possible role of Se and metastasis. 

Metastasis is a multi-step process that begins with the invasion of tumor cells into the adjacent tissue followed by trans-endothelial migration into circulating vessels (intravasation) leading to extravasation into tissues and ending with cell proliferation and subsequent angiogenesis at secondary sites [29]. In this review, we will present the current knowledge of how selenoproteins and Se supplementation may affect tumor migration, invasion, angiogenesis, and overall metastasis in breast, prostate, colon, melanoma, fibrocarcinoma, glioma, skin, liver, and lung cancers. The Se compounds and selenoproteins, which are suggested to affect metastasis are summarized in Table 1 and Table 2. 

**Table 1:** Effects of Se compounds on metastasis (migration, invasion, and angiogenesis).

Se compounds	Studies	Cancer/cells	Function	Reference
MSA	*in vitro*	HUVEC	reduce MMP-2, apoptosis	[9,30]
	*in vitro*	human breast cancer cells, MDA-MB-468 and MCF-7	reduce VEGF	[8]
	*in vitro*	human prostate cancer cells, DU145	reduce VEGF	[8]
	mice	human prostate cancer cells, DU145	reduce tumor growth and angiogenesis	[31]
	rat	rat prostate cancer cells, PAIII	reduce HIF-1α, and VEGF, reduce metastatic lung foci	[32]
	*in vitro*	human fibrosarcoma cell, HT1080	inhibit cell invasion, inhibit MMP-2 activation, reduce MT1-MMP and increase TIMP-2	[9]
	mice	Lewis lung carcinoma cell	reduce lung metastasis, reduce plasma uPAand PAI-1	[33]
	*in vitro*	human clear cell renal cell carcinoma, RC2	reduce HIF-1α, and VEGF	[34]
	*in vitro*	human head and neck squamous cell carcinoma, FaDu	reduce HIF-1α, and VEGF, increase prolyl hroxylases	[35]
MSC	*in vitro*	murine breast cancer cells, TM6	inhibit migration	[36]
	rat	carcinogen-induced breast cancer	reduce angiogenesis	[37]
	mice	human breast cancer cells, MCF-7	reduce angiogenesis	[38]
	mice	human colon cancer cells, HCT-8, HT-29 and GEO	reduce angiogenesis	[39,40]
	mice	human small cell lung cancer, H69	reduce microvessel density, increase vascular maturation	[41]
	mice	human nonsmall epithelial lung carcinimo, A549	increase vascular maturation	[41]
	mice	human head and neck squamous cell carcinoma, FaDu	reduce COX-2, iNOS, HIF-1α, and VEGF, reduce microvessel density, increase vascular maturation, drug delivery and distribution	[35,42,43]
	mice	human head and neck squamous cell carcinoma, A253	reduce microvessel density, increase vascular maturation, drug delivery and distribution	[41]
SeM	mice	murine breast cancer cells, 4T1.2	most protection against metastasis	[44]
	mice	melanoma	reduce lung metastasis	[45]
MeCN	*in vitro*	HUVEC	reduce MMP-2	[9]
methylselenol	*in vitro*	human fibrosarcoma cell, HT1080	reduce cell migration and invasion, decrease expression and activity of MMP-2 and MMP-9, increase TIMP1 and TIMP2	[46]
selenite	*in vitro*	HUVEC	apoptosis	[30]
	*in vitro*	mammaery endothelial cells	reduce VEGF	[47]
	rat	carcinogen-induced breast cancer	inhibit VEGF, reduce angiogenesis	[37]
	*in vitro*	human fibrosarcoma cell, HT1080	reduce cell migration, reduce cell-ECM attachment, reduce MMP-2, MMP-9 and uPA, increase TIMP-1	[48]
	mice	murine melanoma cell, B16BL6	reduce lung metastasis	[49]
	mice	murine melanoma cell, B16F10	reduce lung metastasis	[50]
	*in vitro*	murine melanoma cell, B16F10	inhibit cell migration decrease HIF-1α, VEGF, and IL-18	[51]
	rat	carcinogen-indeced liver cancer	reduce angiogenesis, inhibit angiogenic factors	[41]
	*in vitro*	human astrocytoma cell, IPSB-18	reduce MMPs amd EGFR, increase MMP inhibitors	[52]
selenate	*in vitro*	human breast cancer cells, MDA-MB-231 and MCF-7	enhance epithelial tight junction, inhibit motility and trans-endothelial invasion	[53]
Se-enriched garlic	rat	carcinogen-induced breast cancer	inhibit VEGF, reduce angiogenesis	[37]
high Se isolated soy proteins	mice	murine melanoma cell, B16BL6	reduce lung metastasis	[54]
Se-enriched malt	rat	carcinogen-indeced liver cancer	reduce angiogenesis, inhibit angiogenic factors	[41,55]

**Table 2:** Selenoproteins involved in metastasis.

Selenoproteins	Studies	Cancer/cell	Function	Reference
TXR1	*in vitro*	mammary endothelial cells	TXR1 inhibition reduces VEGF, cell migration, proliferation and tube formation	[47]
	mice	Lewis lung carcinoma cell	TXR1 inhibition reduces lung metastasis	[56]
	*in vitro*	Lewis lung carcinoma cell	TXR1 inhibition reduces HGF and OPN	[56]
	clinically	human oral squamous cell carcinoma	correlated with lymph node metastasis and with the clinical stage	[57]
GPX3	*in vitro*	human prostate cancer cells, PC3, DU145 and LNCaP	GPX3 overexpression reduces cell invasion	[58]
	mice	human prostate cancer cells, PC3 with GPX3 overexpression	GPX3 overexpression reduces primary tumor sizes, eliminates metastasis, and promotes survival	[58]
GPX2	*in vitro*	human colon adenocarcinoma cell, HT29	inhibit cell migration and invasion	[59]
SBP1	*in vitro*	human colon cancer cell, HCT116	SBP1 overexpression inhibits cell migration	[60]
	*in vitro*	human liver cancer cell, SMMC7721	SBP1 reduction increases cell migration and GPX1 activity	[61]
Sep15	mice	murine colon cancer cells, CT26	Sep15 inhibition reduces lung metastasis	[62]

## 2. The Effects of Se on Endothelial Cells

Angiogenesis, the formation of microvessels from existing vessels, is a critical and mandatory step in solid tumor development and metastasis. The distribution of oxygen and nutrients to support tumor growth depends on the completion of these newly created networks of vessels because passive diffusion cannot provide sufficient nutrition once the tumor mass exceeds a certain size [47]. The production of pro-angiogenic cytokines, such as vascular endothelial growth factor (VEGF), by tumor cells and the response to angiogenic signals by endothelial cells are necessary to generate new vessels. Hepatocyte growth factor (HGF), interleukin-1 (IL-1), interleukin-6 (IL-6), interleukin-8 (IL-8), stromal cell-derived factor 1 (SDF-1), growth-regulated peptide-alpha/growth-regulated oncogene-1 (GRO-α), angiopoietins, osteopontin (OPN), and fibroblast growth factors (FGF) promote angiogenesis as well [30]. On the other hand, endothelial cells respond to the angiogenic signals by increasing the production of matrix metalloproteinases (MMPs), such as MMP-2, to degrade the surrounding extracellular matrix, enhancing cell motility for remodeling and invasion, and promoting cell division [47]. Therefore, both tumor cells and endothelial cells are potential targets for inhibition of angiogenesis. 

Using human umbilical vein endothelial cells (HUVEC) to evaluate the anti-angiogenesis activity of a “nutrient mixture”, which contained several antioxidants including Se, Roomi *et al.* [63] demonstrated that the nutrient mixture significantly inhibited cell migration and invasion in a dose-dependent manner. The expression of MMP-2 and the tube formation of HUVEC, a rapid differentiation process in which endothelial cells develop into three dimensional capillary-like structures, were also strongly blocked [63]. Further examination revealed that the inhibition of MMP-2 and the tube formation of HUVEC by Se in endothelial cells was restricted to specific Se compounds [10,63]. While the precursors of methylselenol, MSA and methylselenocyanate (MeCN), effectively inhibited MMP-2 production by reducing MAPK1/2 phosphorylation, the precursor of selenide, selenite, failed to produce the same results [10]. On the other hand, the inhibition of VEGF production in mammary endothelial cells, resulting from selenite supplementation, was specifically related to the activity of TXR [53]. In this study, the reduction of cell migration, proliferation, and tube formation was also negatively modulated by TXR. Furthermore, MSA and selenite induced apoptosis in HUVEC cells [36]. While MSA led to caspase-dependent apoptosis through the p38 MAPK pathway that was partially regulated by ERK1/2 and AKT, selenite induced caspase-independent apoptosis. These data indicated that Se affected cellular function of endothelial cells. However, the role of selenoproteins and mechanisms involved in these processes are still unclear. 

## 3. Se in Breast Cancer

Tight junctions not only control paracellular diffusion and permeability, but also play an important role in the maintenance of cell integrity [37]. In epithelial cells, tight junctions serve as adhesion complexes and keep cells together. In order to metastasize, tumor cells must dissociate from surrounding cells, break the barrier formed by tight junctions, and interact and penetrate the vascular endothelium. Therefore, strengthening tight junctions adds some protection against metastasis development. Martin *et al.* [38] tested the possibility that Se was an anti-metastatic agent using human metastatic MDA-MB-231 and non-metastatic MCF-7 breast cancer cells *in vitro*. They reported that selenate effectively enhanced the trans-epithelial electronic resistance and reduced the paracellular permeability to large molecules in breast cancer cells, which suggested that the cell-cell attachment was increased and the cell colonies were “sealed off” more effectively. Under the influence of selenate, MDA-MB-231 cells displayed very limited motility and showed decreased ability to penetrate endothelial cell layers. MSC also inhibited the migration of murine mammary tumor cells, TM6 [64]. These findings indicated that Se stabilizes cell structure, reduces cell motility, and limits cell migration and invasion in cell culture. 

In addition to affecting endothelial cells and reducing MMP-2 production, methylated Se can also inhibit VEGF expression in breast cancer cells [10]. Jiang *et al.* found that the VEGF reduction in human breast cancer cells, MCF-7 and MDA-MB-468, was MAPK-independent [65]. Nevertheless, this *in vitro* evidence suggested that Se impacted both endothelial and cancer cells and led to the reduction of major regulatory molecules in angiogenesis. An earlier animal study in a carcinogen-induced rat model confirmed that Se reduced angiogenesis. The microvessel density in mammary carcinoma was significantly reduced in rats supplemented with Se-garlic compared to controls [65]. Although the evidence suggested that *in vitro*, selenite could not reduce VEGF expression in breast cancer cells and was unlikely to inhibit angiogenesis [10], *in vivo*, the rats fed selenite, 3 ppm, exhibited a significant reduction of microvessel density [65]. Some mammary tumors from rats fed with Se-garlic and selenite supplementation showed decreased VEGF expression as well (an overall 45% and 75% reduction respectively). Another interesting finding from this study was that Se was able to target established tumors. Furthermore, the effect of Se on angiogenesis inhibition was rapid. Rats with sizeable mammary carcinoma were supplemented with either MSC or selenite for three days. One day after the last treatment, it was found that the microvessel density in both groups decreased about thirty percent. Another *in vivo* study using a MCF-7 xenograft model confirmed the effectiveness of MSC on existing tumors and indicated that MSC was capable of reducing microvessel density in three days [66]. 

Recently, we studied the effect of dietary selenite, SeMet and MSA on breast cancer metastasis in a 4T1.2 mouse model. 4T1.2 cells are a bone metastatic variant derived from a murine highly metastatic breast cancer, 4T1 [31]. When injected into mice orthotopically, 4T1.2 cells can metastasize to several secondary organs, including lung, heart, liver, kidney, and bone. We maintained mice on several Se diets (Se-deficient, 80 ppb selenite as Se-adequate, 400 ppb selenite as selenite-supplemented, 3 ppm SeMet as SeMet-supplemented and 3 ppm MSA as MSA-supplemented) for three months before we injected 4T1.2 cells directly into the mammary gland. We found that although selenite was able to reduce the growth of the primary tumor initially, this protection was lost relatively soon. In contrast, methylated Se showed more prolonged inhibition of tumor growth and SeMet significantly reduced the primary tumor size throughout the experiment. In this highly metastatic model, none of these Se compounds inhibited metastasis completely. Interestingly, we found that selenite supplementation resulted in the most severe metastasis and increased the kidney and bone metastasis incidence. On the other hand, SeMet provided the most protection and generally lowered the metastatic tumor burden in secondary organs [44]. 

In conclusion, Se has been shown to strengthen tight junctions and reinforce cell-cell attachment in breast cancer. It has also been reported that breast cancer migration and invasion were significantly limited by Se *in vitro* [34,35]. These changes may hinder the ability of breast cancer cells to break cellular barriers and initiate metastasis. Angiogenesis was also impaired by the presence of Se. The rapid reduction of microvessel density suggests the possibility of utilizing Se supplementation as an anti-angiogenesis therapy. Methylated Se provided more protection than inorganic selenite against breast cancer metastasis. Our findings emphasized the differences among Se compounds and the urgent need to search for the optimal formulation and dose. 

## 4. Prostate Cancer

Researchers have found in human trials that basal plasma Se levels in the participants are critical to the preventative effect of Se [3,15,32]. A recent dose-response, meta-analysis conducted by Hurst *et al.* concluded that, compared to men with approximately 60 ng/mL Se in the plasma, those with Se concentrations between 135 and 170 ng/mL had a reduced risk of prostate cancer by 15%–25%. Their risk of advanced prostate cancer was reduced even more remarkably by 40%–50% [32]. This greater effect of Se on advanced disease, compared to its effect on localized tumors, was also highlighted in the NPC trial and several other studies [3,58]. These data indicated that Se is likely to interfere with prostate cancer metastasis development. This hypothesis has been supported by several *in vitro* and animal studies. MSA has been shown to reduce VEGF production in human prostate cancer cells, DU145, through a MAPK1/2-independent pathway [10]. Similar to the breast cancer cells, it was noted that the inhibition of VEGF was MSA-specific, and that selenite did not alter VEGF production. The reduction of VEGF may contribute to the anti-angiogenesis effect of MSA noted by Wang *et al.*, in addition to the fact that MSA induced G1 phase arrest in human microvascular endothelial cells, TIME (telomerase-immortalized human miceovascular endothelial cells) [67]. In a DU145 prostate cancer cell xenograft model, MSA effectively reduced tumor growth and intratumoral microvessel density. The anti-metastasis effect of MSA has been reported in a PAIII model of invasive prostate cancer. Two weeks after PAIII cells were injected through the tail vein, rats received daily MSA supplementation for two weeks. This treatment significantly reduced the metastatic lung foci found in the MSA-fed rats [68]. This particular experiment did not address the effect of MSA on primary tumors or the events that happen before cancer cells arrive in the secondary organs. The inability to form detectable metastatic lung foci could be a result of MSA reducing hypoxia-induced factor 1alpha (HIF-1α), a known angiogenesis promoter, and subsequently inhibited its downstream target, VEGF [68].

Other than Se compounds, selenoproteins are also involved in the regulation of metastasis. GPX3 is often down-regulated in prostate cancer cells [60] and is considered to be a tumor suppressor gene [60]. Over expression of GPX3 led to significant reduction of invasiveness in human prostate cancer cells, PC3, DU145, and LNCaP *in vitro* [60]. When GPX3-over expressing PC3 cells were subcutaneously injected into SCID mice, the mice developed smaller primary tumors, and showed no signs of metastasis with remarkable mortality enhancement. Taken together, methylated Se reduced VEGF production and inhibited microvascular endothelial cell proliferation, which contributed to the failure of angiogenesis. GPX3 may be a tumor and metastasis suppressor in prostate cancer. 

## 5. Colorectal Cancer

Selenium-binding protein 1 (SBP1), a Se-containing protein, is a marker for colonic cancer differentiation. A reduction in expression of SBP1 is associated with poor prognosis [69,70]. It was hypothesized that SBP1 may be involved in the regulation of cancer growth and progression. Pohl *et al.* [59] provided evidence indicating that over expression of SBP1 in human colon cancer cells, HCT116, not only reduced cell proliferation, increased apoptosis, but also inhibited cell migration. On the other hand, GPX2 was up-regulated in colorectal cancers [40,62] and appeared to be either beneficial or detrimental based on the stage of cancer development. It was reported that GPX2 supported HT29 human colon adenocarcinoma cell proliferation but inhibited cell migration and invasion *in vitro* [39]. The inhibitory effect of GPX2 appeared to be independent of the anti-oxidant ability. Furthermore, this study suggested that the inhibition was GPX2-specific because an increase in Se, which led to the increase in other selenoproteins, had no effect on cell migration and invasion when GPX2 was specifically knocked-down. Another selenoprotein, which has been studied for its involvement in colorectal cancer, is Sep15. The expression of Sep15 is increased in many colon cancer cell lines. The presence of Sep15 was found to be critical for primary tumor formation and metastasis. Knockdown of Sep15 almost eliminated tumor incidence when Sep15-deficient murine colon cancer cells, CT26, were injected subcutaneously. Moreover, when Sep15-deficient CT26 cells were injected into the tail vein, there were significantly reduced lung metastatic lesions [48]. 

To test whether dietary Se had any effect on colon cancer progression, Bhattacharya *et al.* [45,46] supplemented mice implanted with HCT-8, HT-29, or GEO human colon cancer cells with MSC and measured tumor growth and intratumoral microvessel density. In all three cell lines, MSC supplementation significantly reduced tumor volume and microvessel formation. Interestingly, the authors showed that in a well-vascularized HCT-8 model, MSC significantly enhanced tumor vascular maturation (*p* < 0.001) [46]. This phenomenon was also observed in the HT-29 model, but it was less significant (*p* = 0.02). The increase in vascular maturation induced by MSC further reduced tumor interstitial fluid pressure and improved tumor drug delivery efficiency. 

## 6. Fibrosarcoma

The effect of selenite on fibrosarcoma was mainly evaluated using human fibrosarcoma cells, HT1080. Selenite supplementation significantly reduced cell invasion, but did not affect cell motility *in vitro* [49]. Instead, selenite loosened HT1080 cell attachment to collagen type I and VI, but did not alter cell-cell attachment. The next important step in the metastatic process is degradation of the extracellular matrix. It has been demonstrated *in vitro* that selenite inhibited the expression and activation of several critical proteinases involved in matrix breakdown, including MMP-2, MMP-9, and urokinase-type plasminogen activator (uPA). Moreover, selenite attenuated the increase in MMP-9 resulting from TNFα stimulation and the subsequent activation of MMP-2. Additionally, the natural inhibitor of MMP-9, tissue inhibitor of metalloproteinase 1 (TIMP1) was increased. However, selenate did not have any of these effects on HT1080 cells, which emphasized the importance of using the appropriate Se compound in any given condition. Similar results were reported using organic Se compound, MSA and methylselenol. Park *et al.* [11] reported that MSA inhibited MMP-2 activation, but did not reduce the increase of pro-MMP-9 and pro-MMP-2 caused by 12-*O*-tetradecanoylphorbol-13-acetate (PMA) *in vitro*. In addition to MMP-9, a membrane-type 1 matrix metalloproteinase (MT1-MMP) and TIMP2 are other crucial regulators of MMP-2 activation. MT1-MMP initiated MMP-2 activation and TIMP2 is a natural inhibitor of MMP-2. Although MSA failed to reduce the expression of MMP-9, it decreased MT1-MMP production and enhanced the expression and activity of TIMP2. By changing the MMPs profile in HT1080 cells, MSA effectively inhibited cell invasion stimulated by PMA. It was believed that methylselenol was the main regulator for the inhibition of MMPs [11,54]. Methylselenol treatment exhibited effects similar to those of MSA on the expression and activation of MMP-2, MMP-9, TIMP1, and TIMP2 [54]. The cell migration and invasion of HT1080 cells were also reduced. 

In summary, MSA and selenite, but not selenate, inhibited cell migration and invasion of HT1080 cells. The cell-matrix attachment was weakened by selenite and both MSA and selenite changed MMP profiles and inhibited extracellular matrix breakdown. These data indicated Se reduced the expression and activation of angiogenic factors produced by HT1080 cells, which increased the difficulty to the metastasis development. 

## 7. Melanoma

The possible use of Se to control melanoma metastasis was noted in the 1990s [50,51]. Both dietary selenite and SeMet supplementation significantly reduced metastatic incidence, the numbers of metastatic lung foci, and the tumor size in mice. Not only did supplementation with Se compounds decrease metastasis, but the mixture of high-Se isolated soy proteins demonstrated similar effects [33]. When mice were supplemented with high-Se isolated soy protein, significantly fewer mice developed extensive lung metastasis and the size of the metastases were reduced. Further studies revealed the mechanisms utilized by selenite to inhibit metastasis [71,72]. First, it appeared that murine melanoma cells, B16F10, were more resistant to selenite than other cancer cells. Twenty μM selenite reduced cell viability by a marginal 20%; 40 μM selenite was needed to cause significant cell loss and apoptosis due to G0/G1 cell cycle arrest. Moreover, without affecting cell survival, selenite inhibited cell migration in a dose-dependent manner. HIF-1α, VEGF and IL-18 were all decreased by selenite treatment and were thought to regulate cell migration. IL-18 regulates the expression of HIF-1α, which in turn controls the production of VEGF. In B16F10 cells, IL-18 enhanced cell migration; selenite was capable of counteracting the effect of IL-18. Besides their involvement in cell migration, HIF-1α and VEGF were essential for angiogenesis. By reducing these two factors, selenite could lessen the angiogenesis signals from melanoma cells and affect primary and metastatic tumor development. However, the concentration of selenite used in these studies was so high that it may be difficult to achieve such levels via dietary means. 

## 8. Lung Cancer

It has been reported that Se-enriched yeast reduced metastasis in a Lewis lung carcinoma mouse model [56]. Although SeMet was the major Se species in Se-enriched yeast [57], it may not be the primary regulator of lung cancer metastasis. Using Lewis lung carcinoma cells, MSA alone caused a reduction of lung metastasis by 55% in the intramuscular injection model and a 20% decrease in the subcutaneous injection model. SeMet supplementation only resulted in a non-significant 30% reduction in the intramuscular model and did not have any effect in the subcutaneous model [41]. MSA, but not SeMet, reduced the expression of uPA and plasminogen activator inhibitor-1 (PAI-1). These findings suggested that MSA may reduce Lewis lung cancer metastasis by inhibiting the entire urokinase plasminogen activator system, which resulted in the decrease of the uPA-mediated extracellular matrix degradation and impaired invasion. Furthermore, MSC has been shown to affect angiogenesis [55]. In a H69 human small cell lung cancer xenograft model, MSC treatment effectively reduced the microvessel density and increased vascular maturation. The increase of vascular maturation can also be observed in an A549 human nonsmall epithelial lung carcinoma xenograft system. However, the effect of MSC on interstitial fluid pressure and drug delivery efficiency was not significant. 

Although TXR1 negatively regulated cell proliferation, migration and the production of several angiogenic factors, such as VEGF and VEGF receptor, in mammary endothelial cells [53], TXR1 was positively associated with the tumorigenicity and metastasis development of Lewis lung cancer [61]. At least two molecules involved in angiogenesis, metastasis, and tumor growth, HGF and OPN, were significantly reduced in Lewis lung cancer cells when the expression and activity of TXR1 was inhibited. TXR1 is over expressed in many cancer cell lines [53,61]. It is important to determine whether Se supplementation increases TXR1 activity and accelerates tumor progression in other cancer systems. 

## 9. Other Cancers

There are only a few reports regarding the relationship between Se and metastasis in other cancers. For instance, an increase in TXR1 expression has been correlated with regional lymph node metastasis and with the clinical stage in oral squamous cell carcinoma [73]. It was hypothesized that the increase of TXR1 promotes tumor aggressiveness and contributes to poor prognosis, suggesting that optimal redox status could play a prominent role in modulating tumorigenesis. In a carcinogen-induced hepatocarcinoma rat model, Se-enriched malt was found to inhibit angiogenesis at least partially through reducing several angiogenic factors, including TNFα, nitric oxide, nitric oxide synthase, IGF II, VEGF, and protein kinase Cα [52,74]. Selenite supplementation produced a smaller effect, which may have been the result of insufficient reduction of angiogenic factors.

In liver cancer, it has been suggested that SBP1 plays a role in metastasis [34]. SBP-1 is a Se-containing protein and its main function is Se transport. SBP1 is highly expressed in normal liver tissue but was nearly non-detectable in highly metastatic liver cancer cell lines. The reduction of SBP1 increased cell migration and GPX1 activity, and the combination of decreased SBP1 and enhanced GPX1 activity could be observed in liver cancer patients with macrovascular invasion. SBP-1 is a downstream target of HIF-1α; therefore, the increase of HIF-1α after hydrogen peroxide stimulation led to the increase in SBP1. Interestingly, in the absence of SBP1, hydrogen peroxide no longer enhanced HIF-1α, which suggested a negative feedback mechanism between SBP1 and HIF-1α. Taken together, loss of SBP1 enhanced cell migration and GPX1 activity. Enhanced GPX1 resulted in a decrease in hydrogen peroxide and other reactive oxygen species, which may lead to the inhibition of HIF-1α. 

In an *in vitro* study, MSA not only inhibited the proliferation of human clear cell renal cell carcinoma, RC2, but also decreased the level of HIF-1α, and its subsequent target, VEGF [35]. The data suggested that the post-translational degradation of HIF-1α was modified by MSA in a proteosome-independent, prolyl hydroxylase-dependent manner. 

In head and neck squamous cell carcinoma, several reports suggest the effectiveness of MSA and its precursor, MSC, for anti-angiogenesis treatment. Under hopoxia, Chintala *et al.* [43] showed that MSA dose-dependently inhibited HIF-1α and VEGF, and increased prolyl hydroxylases in FaDu human head and neck squamous cell carcinoma cells *in vitro*. These findings indicated that MSA affected the level of HIF-1α through the post-translational degradation. Unlike RC2 cells, the degradation of HIF-1α by MSA in FaDu cells was proteosome-dependent [35]. *In vivo*, the effect of MSC on angiogenesis has been demonstrated in a FaDu xenograft model [42,43,75]. Yin *et al.* [75] suggested that MSC treatment efficiently reduced cyclooxygenase-2 (COX-2), inducible nitric oxide synthase (iNOS), and HIF-1α expression. Besides HIF-1α, COX-2 and iNOS are also important angiogenesis regulators. NO generated by iNOS stimulates the expression of COX-2 which is the key enzyme for prostagladins (PGs) production. One of the PGs, PGE2, promotes angiogenesis by increasing VEGF. By inhibiting the expression of HIF-1α, COX-2, and iNOS, MSC significantly reduced the expression of VEGF [43]. Overall, the net effect of MSC on angiogenesis has been clearly demonstrated by Bhattacharya *et al.* [42] and Rustem *et al*. [55] using FaDu and A253 (human head and neck squamous cell carcinoma) xenograft models. The MSC treatment significantly reduced the microvessel density and increased vascular maturation, which contributed to the enhanced delivery efficiency and distribution of the anti-cancer drug, doxorubicin. 

Selenium is distributed throughout the body and reported to be particularly well-maintained in the brain and testis even after a prolonged period of Se deficiency [76]. Rooprai *et al.* [77] demonstrated that selenite induced apoptosis in human astrocytoma cells, IPSB-18, derived from a biopsy of an anaplastic astrocytoma. Their data also suggested extensive changes in the expression of MMPs. MMP-2, -9, 14, -15, -16, -24 were reduced as were their inhibitors, TIMP-1, -2, -3, -4. Epithelial growth factor receptor (EGFR) is often over expressed in glioma, and contributes to cell proliferation, survival, motility, and invasion [78]. It was reported that selenite decreased its expression. In contrast, it was noted that MMP-25 was elevated by selenite. Taken together, the anti-metastatic effect of selenite on glioma was likely due to two mechanisms: apoptosis induction and an alteration in the profiles of several MMPs. 

## 10. Conclusion

### The Future Role of Se in Cancer Metastasis

Although epidemiological data and clinical trial results do not fully agree on the benefits of Se supplementation, the results of many *in vitro* and animal studies have suggested that Se is a promising chemopreventive and anti-cancer agent. Criticisms and corrections were made in the post-SELECT era to address many questions that were previously neglected. For instance, the optimal Se concentration, which exhibits a particular protective effect, may be very limited. In addition, it is constantly a challenge to determine the most effective Se compound since various Se compounds may affect cells through different mechanisms. In contrast to many studies aimed at cancer prevention and treatment of primary tumors, much less work has been done with cancer progression and metastasis. Recently, more researchers have noted the value of Se as an anti-metastasis agent (including anti-migration, anti-invasion, and anti-angiogenesis). In this review, we have summarized the current understanding of the effect of Se on metastasis in several cancers. Some cancers have drawn much of the attention, and the anti-metastasis effect of Se was more or less established in these cancers. For instance, the results of some studies demonstrated that Se affected cell-cell attachment, migration, and angiogenesis in breast cancer. In order to design a Se-based drug to treat breast cancer metastasis, it is urgent to study how Se affects metastasis development at the cellular and molecular level to establish possible mechanisms. On the other hand, there is limited data to suggest the involvement of Se or selenoproteins in the metastasis development of some cancers such as glioma. In these cases, more data from *in vitro* and animal studies are needed to solidify the relationship between Se and metastasis.

In our own studies with breast cancer *in vitro* and *in vivo* we also revealed that the inhibitory effect of Se on metastasis might be specific to certain forms of Se [44]. It is important to understand why only certain compounds exhibited the effect of anti-metastasis, but not others. Understanding the differences among various forms of Se not only improves our knowledge of Se biology, but also helps us to understand how micronutrients regulate cellular functions. Identification of specific selenoproteins that may control metastasis in specific cancers would provide good targets for therapy. In summary, although further research needs to be performed to understand the precise mechanisms, Se has been shown to affect cancer cell migration and invasion, inhibit angiogenesis, promote vascular maturation, enhance drug delivery and distribution, and decrease metastasis. This review highlights the potential application of Se in cancer metastasis prevention/treatment. 
